# Vale! 1887

**Published:** 1887-12

**Authors:** 


### VALE! 1887.

With this number Volume VIII is brought to a close. We can, we think, look with pride upon what has been accomplished, and anticipate yet better things for the future. "Experientia does it," and the publishers certainly are better prepared to furnish a valuable journal now than they have been at any time in the past. All we ask for is the support of those for whom we labor. The many letters of high commendation received only act as an incentive to yet greater exertions. Not one such but has been gratefully read, and if we have not made individual acknowledgment it was because of lack of opportunity. We are fain to believe that there is a personal attachment between the publishers of this Journal and its readers, and it is our earnest desire that this feeling shall become yet warmer. The editor is well aware of his shortcomings, and that sometimes his course may not be endorsed by all his readers, but he trusts they have faith in the honesty of his intentions, that they believe he is conscientiously laboring for what he thinks is the greatest good of our beloved profession, and that they will excuse the errors which are of the head and not of the heart.

It has been our pride that very seldom is a subscriber lost to the INDEPENDENT PRACTITIONER, and we trust that every one will renew for the coming year, and thus help us to be of use to them in return. Bills will be sent out with the January number, and we bespeak for them prompt attention. We do not desire to press old subscribers to their inconvenience, but shall hope that our dues will not long be withheld. Receipts for money sent us will be enclosed in the number for the month succeeding the reception.

We desire to call attention to the very liberal offer made in the prospectus. Our main object in sending out Prof. Stowell's book at the almost nominal price for which it is furnished, is that it may act as an educator. No one can look at its beautiful plates without obtaining a distinct general knowledge of the internal structure of the human tooth. We lose money on every copy of it furnished, and our only hope of complete reimbursement lies in the expectation of an increased subscription list. Under these circumstances, all will comprehend that the rule that the book can only be furnished in connection with a subscription must be inflexible, and that the money in such cases must accompany the order. We reach the extreme limit of ability in making the offer, and hope that this fact will be appreciated, and that we shall not be asked to do that which is impossible.

And now, permit us in the most earnest and heartfelt manner to extend to every reader all the compliments of the Merry Christmas season, and to express the hope that the coming year will mark a great advancement in every good and desirable thing. At the close of the year 1888, may we all be better, richer, happier than now. Written as this is upon the glad Thanksgiving Day, we can but exclaim with Tiny Tim, "God bless us, every one."

